# Effector-Dependent and -Independent Molecular Mechanisms of Soybean–Microbe Interaction

**DOI:** 10.3390/ijms232214184

**Published:** 2022-11-16

**Authors:** Jinhui Wang, Hejia Ni, Lin Chen, Jianan Zou, Chunyan Liu, Qingshan Chen, Pascal Ratet, Dawei Xin

**Affiliations:** 1 Key Laboratory of Soybean Biology in Chinese Ministry of Education, College of Agriculture, Northeast Agricultural University, Harbin 150030, China; 2 CNRS, INRAE, Univ Evry, Institute of Plant Sciences Paris-Saclay (IPS2), Université Paris-Saclay, 91190 Gif sur Yvette, France; 3 Institute of Plant Sciences Paris-Saclay (IPS2), Université de Paris, 91190 Gif sur Yvette, France

**Keywords:** soybean, interaction, cyst nematode, *Pseudomonas*, *Xanthomonas*, rhizobium

## Abstract

Soybean is a pivotal staple crop worldwide, supplying the main food and feed plant proteins in some countries. In addition to interacting with mutualistic microbes, soybean also needs to protect itself against pathogens. However, to grow inside plant tissues, plant defense mechanisms ranging from passive barriers to induced defense reactions have to be overcome. Pathogenic but also symbiotic micro-organisms effectors can be delivered into the host cell by secretion systems and can interfere with the immunity system and disrupt cellular processes. This review summarizes the latest advances in our understanding of the interaction between secreted effectors and soybean feedback mechanism and uncovers the conserved and special signaling pathway induced by pathogenic soybean cyst nematode, *Pseudomonas*, *Xanthomonas* as well as by symbiotic rhizobium.

## 1. Introduction

Soybean is one of the world’s four main grain crops, along with wheat, rice, and maize and is the only legume in that set [1]. In addition to interacting with pathogens, soybean can also develop symbiotic relationships with rhizobium. During the interaction between soybean and microorganisms, a sophisticated signaling communication is required [2,3]. Microorganisms have developed numerous ways of transporting cargo protein between locations, which largely involve the assistance of specific protein secretion systems [4,5,6] that are used in an array of processes and are essential for the growth of bacteria, fungi, and nematodes during infection [5]. Molecules secreted by pathogens to help infection are called effectors and are secreted by different (at least six) secretion systems associated with different functions. Secreted effectors can be recognized by the host immunity receptors and can have positive and negative effects on the plant–microbe interaction [5,6]. The type III secretion system (T3SS) is mostly associated with pathogenicity in the plant–microbe interactions. Type III effectors (T3Es) are injected into host plant cells by the T3SS ‘syringe’-like core, hijack the immunity system of the host, bind the plant defense proteins, make them inoperant [7,8,9], and promote the progression of pathogen virulence [10,11]. During symbiosis, rhizobium T3Es are also secreted into the soybean host cell by a T3SS, in which they play an essential role in the establishment of symbiosis [12,13,14,15,16]. Nematodes use their stylets to secrete molecules, known also as effectors, into the host tissues or cells, targeting important host molecular components/pathways to facilitate parasitism [17]. 

Molecular-pattern-triggered immunity (PTI) and pathogenic-effector-triggered immunity (ETI) are two interconnected defense strategies of plant responses to microorganisms [18]. Pattern-triggered immunity (PTI) refers to the first layer of the plant immune system that is triggered by molecular patterns of microorganisms recognized by *Pattern Recognition Receptors (PRRs)*. For effector-triggered immunity (ETI), the host recognizes the microorganism effectors generally by NLR receptors and the recognition response is associated with the long-standing gene-for-gene hypothesis [19]. Facing the multiple functions of the effectors, the host also developed multiple immunity strategies, including effector-triggered susceptibility (ETS) and effector-triggered immunity (ETI) [20,21]. For bacteria, the only proteins known to induce ETI are T3Es [22]. 

In this review, we will focus on the general function of effectors derived from symbiotic bacteria (rhizobium), pathogenic bacteria (*Pseudomonas*, *Xanthomonas*), and soybean cyst nematode (*Heterodera glycines Ichinohe*) that are necessary for infection. The effector response genes of soybean will also be reviewed. 

## 2. Interaction between Nematode Effectors and Soybean *R* Genes 

Soybean cyst nematode (SCN, *Heterodera glycines Ichinohe*) disease is one type of globally classified and devastating *Glycine max* disease [23,24,25]. SCN is a mixed population in the field and exists in multiple physiological subspecies [26,27,28]. The most cost-effective method for controlling SCN is the combination of resistant varieties and non-host varieties rotation, but due to the monoculture of soybean due to the nature of the soil, crop rotation can be limited. In addition, highly toxic effective chemical nematicides are now banned or restricted [29,30]. At present, the resistance of most resistant germplasms is singular and the number of resistant germplasms against nematodes is more limited [31]. The resistance against nematodes previously developed in breeding lines has been reduced or lost due to the rapid evolution of these animals [31,32]. The unknown feature of the protection mechanism and/or of the resistance makes these resources unusable. It is, thus, urgent to analyze the resistance mechanisms so that these resistant germplasm resources can be fully utilized and molecular-assisted breeding accelerated.

**Figure 1:**
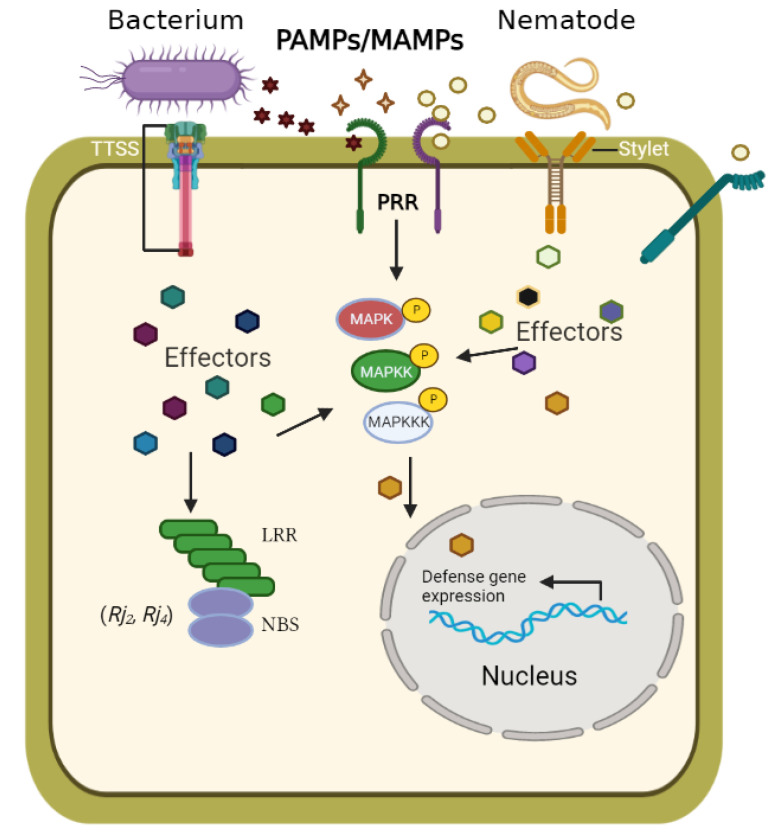
Schematic of soybean immunity response to effectors. Pathogens and rhizobium of all lifestyle classes (color and labeled) express PAMPs (pathogen-associated molecular patterns) and MAMPs (microbe-associated molecular patterns) as they colonize soybean (shapes are color coded to the pathogens and rhizobium). Soybean perceives these via extracellular PRRs and initiates PRR-mediated immunity (PTI). Pathogens and rhizobium deliver effectors to both the soybean cells and cell apoplast to block PAMP/MAMP perception (not shown). These effectors are addressed to specific subcellular locations where they can suppress PTI and facilitate virulence and symbiosis. Intracellular NLR receptors can sense effectors. It is not yet clear whether each of these activation modes proceeds by the same molecular mechanism, nor is it clear how, or where, each result in NLR-dependent effector-triggered immunity (ETI). (Modified from Okazaki and Dangl [5,63]).

To invade the host without triggering defense reactions, SCN secretes a diverse set of proteins called effectors, interacting with the plant immunity signaling [33,34,35]. In SCN, about 48 effectors genes have been identified, 40 effectors showed evidence of novel structure variants, and 11 effector genes contain phenotype-specific sequence polymorphism [26,35,36]. The biological function or targets of most of these effectors of SCN still have to be described (Figure 1). Only a few of the host genes that can interact with these nematode effectors were identified. For example, potato Gpa2 can recognize the effector Gp-RBP-1 of *Globodera pallida* [37] and tomato Cf-2 can trigger defense-related programmed cell death in plant cells when recognizing Gr-VAP1 of *Globodera rostochiensis* [38]. Cell-wall-degrading effectors have been described, but only a few of them were shown to contribute to the de novo organogenesis and maintenance of feeding sites [33]. *Meloidogyne incognita* PASSE-MURAILLE (MiPM) (a small pioneer protein predicted to contain a secretory peptide that is up-regulated mostly in the J2 parasitic stage) can interact with the CSN5 Subunit of the COP9 Signalosome of soybean [39]. HgSLP-1 of *Heterodera glycines* can interact with *Rhg1* α-SNAP physically, possibly functioning as an avirulence protein. When absent, it helps SCN to evade host defenses [40]. 

Resistant resources, such as Peking, PI88788, Fayette, and PI437654, were identified by screening for resistant germplasms. These germplasms all have the *Rhg1* locus, which is a QTL (Quantitative Trait Locus) mapping to linkage group G (Chromosome 18) [41,42,43]. The single resistant locus segment is about 31.2 kb and contains genes *Glyma.18g022200* (*Glyma.18g02580*), *Glyma.18g022500* (*Glyma.18g02590*), and *Glyma.18g022700* (*Glyma.18g02610*) that show higher expression in root from SCN-resistant compared to SCN-susceptible varieties [44]. *Glyma.18g022500* codes an α-soluble N-ethylmaleimide-sensitive factor attachment protein, named α-SNAP18. Copy number variation in these germplasms can underly the resistance to SCN directly. For example, at least two copies are required to make the germplasm resistant to SCN [42,45,46]. In plants containing the *Rhg1* resistance type, α-SNAP depletes the abundance of SNARE-recycling 20S complexes, disrupts vesicle trafficking, induces an elevated abundance of NSF (N-ethylmaleimide-sensitive factor), and causes cytotoxicity [44]. The difference in SNPs located in the *GmSNAP18* could be used to identify the SCN-resistant and SCN-susceptible germplasms. *Rhg4* is also a locus underlying SCN resistance [44]. *Glyma.08g108900* codes a serine hydroxymethyl transferase (SHMT08) [47,48] and was identified as the responsible gene in the *Rhg4* locus. SHMT plays a role in one-carbon metabolism, methionine synthesis, and the maintenance of redox homeostasis during photorespiration [49,50,51]. Copy number at this locus is also important for resistance.

In general, genes conferring resistance against pathogens regulate plant defenses. Signaling and transduction networks were also shown to play pivotal roles in plant resistance against nematodes [52]. However, novel mechanisms for resistance against SNC were identified in soybean [53]. In the resistant germplasm PI 88788 and Peking, the copy number of rhg1 is more than 5.6 while the resistance of soybean to SCN seems to be independent of the *Rhg4 GmSHMT08* haplotype [45,54]. Once the *GmSNAP18* copies dropped below 5.6, a ‘Peking’-type *GmSHMT08* haplotype (*rhg4* locus) was required to ensure the SCN resistance [26,54]. This suggests that there is epistasis and haplotype compatibility, copy number variation, promoter variation, and network regulation mechanisms that regulate soybean resistance to SCN [54]. In addition to *Rhg1* and *Rhg4*, other genes underlying soybean resistance to SCN were found on the different chromosomes [23], all chromosomes except chromosome 02 [55]. Some genes could only be identified in special genetic background while some single nucleotide polymorphisms (SNPs) only exist in special germplasms [56,57]. This also supports that the resistant genes and fragments are distributed in special germplasm, such as Peking, PI494182, Pingliang, and PI88788 [26]. In the wild soybean, novel loci and SNP were also identified by genome-wide association study (GWAS) and QTL mapping [23]. 

To further elucidate the molecular mechanism of soybean resistance to SCN, the interaction network of α-SNAP was identified. Genes *Syn12* and *Syn16* belong to the syntaxins of the t-SNARE family and can interact with *Rhg1* α-SNAP [58]. *Rhg1 WI12* (*Glyma.18G02270*) can interact with *DELLA11* (*Glyma.11G216500*) directly. The expression of *WI12* was induced by JA (jasmonic Acid), SA (salicylic acid), and GA (gibberellic acid) [59]. Interestingly, the JA and SA content increased concomitantly with the soybean resistance to SCN and soybean resistance to SCN can be decreased after the GA treatment. Increasing the JA content triggers the degradation of JAZ1 to liberate DELLA, which promotes a defense response [60]. JA and SA have a direct interplay with each other, with JA signaling controlling SA level [28]. Reciprocal antagonism between the JA and GA pathways is a key mechanism enabling plants to balance growth and defense [61]. JA and SA can interfere with GA metabolism and stabilize DELLA, while increasing GA concentration stimulates the interaction of DELLA with SCF^SLY1^ complex. Once recruited to SCF^SLY1^, DELLA is polyubiquitylated and then subsequently degraded [62]. WI12 can also regulate the expression of DELLA positively while knockout of *DELLA11* results in decreased levels of JA and SA [59]. While *GmSHMT* can interact with *GmPR08-Bet VI*, the interaction was enhanced when *GmSNAP18* was also present [54]. This suggests that the interaction between *Rhg1* and *Rhg4* might be in a complex composed of *GmSHMT08*/*GmSNAP18*/*GmPR08-Bet VI* [54,59].

## 3. Function of *Pseudomonas* Effectors in Soybean–*Pseudomonas* Interaction

**Figure 2:**
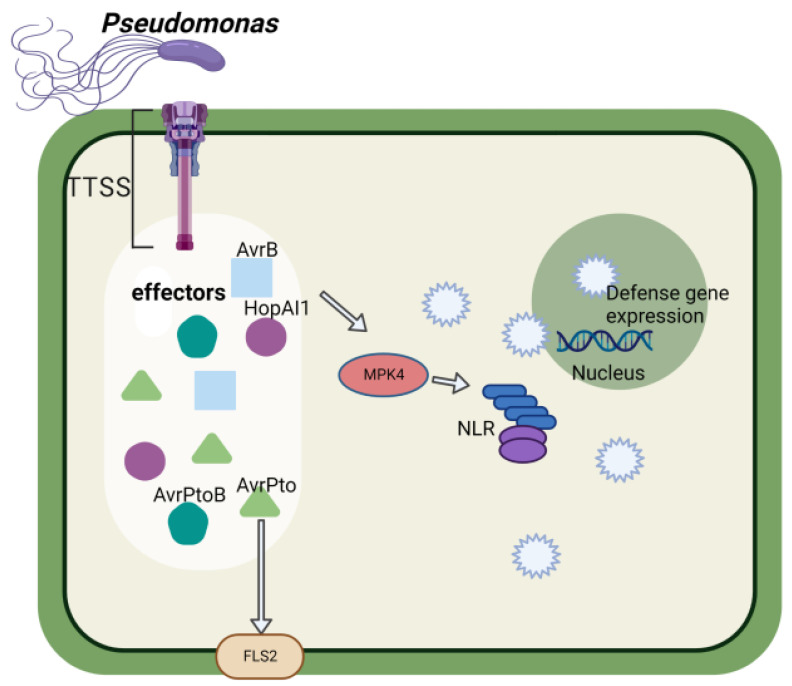
Schematic diagram of soybean immune response to effectors secreted by *Pseudomonas*. *Pseudomonas* secretes effectors into soybean cells to promote virulence and symbiosis. These effectors enhance plant susceptibility by activating functional intracellular proteins such as MPK4, which trigger NLR-dependent effector-triggered immunity (ETI).

*Pseudomonas syringae* is a plant pathogen that can colonize the intercellular spaces of leaves and other aerial organs. *P. syringae* strains are classified into about 50 pathogenic bacteria, mainly according to the origin of the host [64]. The amino acid sequences of flagellin from *Pseudomonas syringae pv*. tabaci and *P. syringae pv. glycinea* are identical, but the ability of these two strains to induce a hypersensitive response in tobacco and soybean is different [65]. The study of *P. syringae* effectors has gone through several phases. First, a small number of effector proteins was identified as “Avr” (Avirulence) proteins in test plants with matching *R* genes [66]. Second, there was a fundamental understanding of the mechanism by which effectors induce ETI by modifying host proteins that are “protected” by NLR (Nod-like receptor) proteins [67]. Third, the effector library of Pto DC3000 and some other strains was identified by bioinformatics and functional screening of the Hrp (hypersensitive response and pathogenicity) promoter [68]. Fourth, a fundamental understanding of the molecular mechanisms by which effectors differentially inhibit PTI or ETI [69,70] has been achieved. The *Pseudomonas syringae* effector AvrPto binds receptor kinases PRR (pattern-recognition receptor), including *Arabidopsis thaliana* FLS2 (Flagellin sensitive2) and EFR (EF-TU receptor) and tomato LeFLS2 to block immune responses in plant cells [71] (Figure 2). The *P. syringae* effector protein AvrB, which is delivered into the plant cell by the bacterial T3SS, interacts with MPK4 (mitogen-activated protein kinases 4) and HSP90 (heat shock protein 90) partners, inducing MPK4 activation in an HSP90-dependent manner, interfering with hormone signaling and enhancing plant susceptibility [72]. However, the inactivation of MPK4 by *P. syringae* effector HopAI1 activates the nucleotide-binding leucine-rich repeat (NB-LRR) protein SUMM2-mediated defense responses [73]. The MAPKs MPK3 and MPK6 are direct targets of HopAI1 [74]. Inhibition of MAPKs by HopA1 inhibits two independent downstream events, enhancement of cell wall defense and transcriptional activation of PAMP-responsive genes. The *P. syringae pv*. tomato AvrPtoB effector has a carboxy-terminal domain that is an E3 ubiquitin ligase [75] and promotes bacterial virulence. Concurrent studies have demonstrated that the phosphorylation of AvrPtoB by *Arabidopsis* SnRK2.8 is required for bacterial virulence [76]. BAK1 is a physiological target of AvrPto, AvrPtoB, and HopF2 with HopF2 targeting BAK1 (Brassinosteroid-insensitive-1-associated receptor kinase-1) to inhibit multiple MAMP signaling at the plasma membrane [77].

AvrB can also suppress flg22-induced callose deposition and basal defense responses [78,79] and it enhances the growth of *P. syringae* on soybean-susceptible cultivars. The virulence activity can be blocked by specific amino acid substitutions at Thr125Ala, Arg266Gly, and Asp297Ala [80]. *P. syringae* effector AvrRpt2 can inactivate the *GmRIN4* (*Glycine max* resistance to *Pseudomonas syringae* pv. maculicola 1 (RPM1)-interacting protein 4) gene homolog and blocks the recognition of AvrB by soybean [81]. T3SS-secreted cysteine protease AvrPphB inhibits immune system activation by blocking the phosphorylation of *GmRIN4* at T198 [82]. Soybean is a natural *P. syringae* host and *GmHID1* (2-hydroxyisoflavanone dehydratase from *G. max*) is a virulence target of *P. syringae* essential bacterial virulence protein HopZ1, promoting *P. syringae* infection of soybean [72]. The PR5 (Pathogenesis-related-5), such as protein GmOLPc (*G. max* alkaline isoform of the PR-5 protein), purified from soybean hull can inhibit the growth of *P. syringae pv. Glycinea* [83]. 

Similarly to previous studies, silencing GmFLS2 not only compromises the resistance of soybean plants to *P. syringae* pv. glycinea, but also attenuates the activation of GmMPK3/6 in response to P. syringae pv. glycinea infection [84]. NopT of Sinorhizobium sp. NGR234 is an effector protease related to the Pseudomonas effector AvrPphB. The NopT protease activity negatively affects nodulation of smooth crotalaria (Crotalaria pallida) [85].

Plant-resistance (R) proteins sense specific pathogen effectors to activate plant defenses and limit pathogen invasion. Soybean cultivars expressing the disease resistance gene *GmRPG1* (Resistance to *P. syringae* pv glycinea) are resistant to *P. syringae* strains expressing the avirulence gene *avrB* [86]. In addition, Keen found that the resistance gene *Rpg4* may partly explain the resistance of soybean to *P. syringae pv* tomato and other *avrD*-carrying pathogens [87]. Two orthologs of the *RPS2* (Resistance to *P. syringae*) gene encoding a CNL (coiled-coil NBS-LRR) domain protein from wild soybean confer resistance to *P. syringae* [88,89]. 

## 4. The Infection Molecular Signaling of *Xanthomonas* on Legume

**Figure 3:**
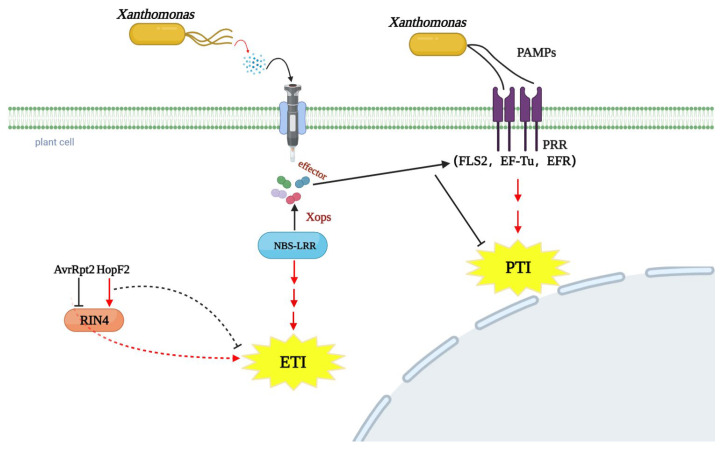
*Xanthomonas* uses the type III secretion system (T3SS) to secrete effectors into host plant cells. When *Xanthomonas* infects plants, the plant surface pathogen recognition receptor (PRR) triggers the PTI response. In response to further infection by pathogens, plants evolved a nucleotide-binding site leucine-rich recombinant protein (NBS-LRR) to recognize the Xops effector. When the effectors are recognized, the plant second line of defense ETI immune response is activated and the plant HR response is triggered.

*Xanthomonas* is a Gram-negative, aerobic, chemo-organotrophic phytopathogen [90]. The cells are straight and rod shaped and the size is 0.4–1.0 μm × 1.2–3.0 μm. Most of the strains secrete insoluble yellow pigment and form yellow pigmented colonies on a solid medium [91]. The word *Xanthomonas* is a combination of the Greek yellow ‘Xanthus’ and the unit ‘monas’, which translates as ‘yellow monad’. The *Xanthomonas* name was originally proposed by Burkholder in 1930 and created by Dowson in 1939. Pathogens in this genus are often associated with plant diseases [92]. Common diseases found in nature include vascular blight, canker, leaf spot, fruit spot, and fusarium wilt. The disease can cause more than 400 different plant hosts, which includes 124 monocotyledonous and 268 dicotyledonous species [93,94]. When *Xanthomonas* infects plants, the plant PAMP (pathogen-associated molecular pattern)-triggered immunity (PTI) and effector-triggered immunity (ETI) are triggered to prevent initial pathogen invasion [95,96,97]. Most PTIs are triggered by PRRs on the surface of plant cells [72,85]. Among them, the most intensively studied are the receptors for flagellin and for the elongation factor Tu (EF-TU) [71,84] (Figure 3). 

So far, at least six protein secretion systems have been found in *Xanthomonas* and the composition and function of type I to type VI systems, as well as the secretion substances, are significantly different [98]. The type III secretion system (T3SS) highly conserved in plant pathogens is very important for the pathogenicity of *Xanthomonas* [99,100] and is responsible for many *Xanthomonas* infections. Pathogenic *Xanthomonas* uses T3SS to influence the pathogenicity of *Xanthomonas* against the host [101,102]. Adhesion factors mediate bacterial attachment to host cells and promote bacterial infection at different stages of the plant [86].

There are 53 known effector families in *Xanthomonas*, which are called *Xanthomonas* outer-membrane proteins (Xops) [86,103,104]. Except for AvrBs1, AvrBs2, and AvrBs3, the remaining effectors are recognized by the corresponding R proteins of the host to trigger plant effector-triggered immunity (ETI) [105]. Previous studies have shown that the effectors XopD and XopN promote the virulence of *Xanthomonas* and the pathogenicity against tomato. XopD binds DNA in the plant nucleus to inhibit the transcription of senescence and defense genes and results in the attenuation of SA-dependent senescence in tomato [106]. Further study of XopD revealed that it could directly recognize SIERF4, a transcription factor that regulates ethylene response in tomato, and inhibit ethylene production [107]. The discovery of this key role for ethylene in plant resistance to *Xanthomonas* helps with understanding the molecular mechanism of plant resistance against *Xanthomonas*. Compared with the pathogenicity of XopD mutant, the pathogenicity of XopN-deficient strains is greatly reduced [108]. This situation not only occurred when *Xanthomonas* infected tomato, but also significantly reduced the virulence when the XopN homolog mutant infected radish [109]. *Xanthomonas* effectors are widely distributed and have certain pathogenic abilities in different species. In rice, the *Xanthomonas* effectors XopN, XopQ, XopX, and XopZ can inhibit the immune response induced by cell-wall-degrading enzyme treatment. XopQ and XopX interact with each other and can also induce the rice immune response [110]. XopP targets the rice E3 ubiquitin ligase OsPUB44 to inhibit resistance to *Xanthomonas* in rice [111]. With further study, researchers have found that some effectors of *Xanthomonas* have transcription activator-like (TAL) activity. Among non-TAL effectors, XopAF and AvrBs2 are the most representative ones, with AvrBs2 being the first reported non-TAL effector [112], essential for host pathogenicity. Studies in tomato and rice found that AvrBs2 promotes bacterial proliferation in plants by inhibiting plant defense responses [113,114]. As a representative of TAL, the AvrBs3 family is the best studied type III effector protein. Multiple members of the AvrBs3 family have toxic functions [115]. AvrXa7, PthXo, and other AvRBS3-like proteins derived from Rice bacterial blight (*Xanthomonas oryzae* PV. Oryzae, Xoo) strongly promote bacterial growth and the formation of necrotic spots in rice [116,117]. Another major TAL effector, PthXo1, regulates the expression of the rice gene Os8N3. When Os8N3 is silenced, plants become resistant to PthXo1 [118].

Although *Xanthomonas* effectors are well described in rice and tomato, there is little information on the effectors that play a major role in the pathogenic properties of soybean *X. axonopodis pv. glycines*. So far, 169 strains of *X. axonopodis pv. glycines* have been identified and divided into three races, each of which carries five to six TAL effectors. In soybean, GmLOB1 confers resistance to Race 2, which is the most pathogenic one, but its connection with TAL-type effectors needs further analysis [119]. In addition, avrXg1, an avrBs3 homolog, was identified in Race 3. The hypersensitivity (HR) of Williams 82 soybean cultivar inoculated with *Xanthomonas* was caused by avrXg1 [120]. This finding provides the research basis for understanding the defense mechanism of soybean against *Xanthomonas* virulence and provides new ideas for seeking practical methods to control soybean bacterial pustules in agricultural production.

The main epidemic disease on the commercial crop soybean is soybean bacterial pustules caused by *X. campestris pv. glycines* (*Xcg*), which were renamed *X. axonopodis pv. glycines* (*Xag*) in 1985 [121]. The symptoms of bacterial pustule disease are the formation of light-green spots and brown pustules surrounded by yellow halos on the surface of soybean leaves, leading to early defoliation [122,123]. Due to early leaf loss, the grain cannot complete the filling period, normally resulting in plant yield reduction up to 40 percent in severe cases [124]. Several virulence factors have been identified in *Xag,* including extracellular cellulase, protease, endo-β-1, 4-mannanase, and pectin acid ligase. They can stimulate the degradation of cellulose and hemicellulose in host plants and have varying degrees of impact on soybean production [125,126,127]. To resist *Xag* invasion, soybean produces isoflavones, such as daidzein, genistein, and glycitein [128]. Recent studies have shown that during the growth and development of soybean, genistein enhances the plants resistance to *Xag* by inhibiting the tyrosinase pathway. Exogenous application of genistein decreases the expression of the virulence factor yapH and promotes the up-regulation of several soybean defense genes [129]. The outer-membrane protein A (OmpA) is very important for the pathogenicity of *Xag*. When OmpA is mutated, the contents of cellulase and pectin acid ligase of *Xag* are significantly reduced and the pathogenicity decreases [130]. In addition, *Xag* virulence genes have also been identified [131,132,133,134,135,136]. This research lays the foundation for understanding the biology of *Xag*, determines its pathogenic cycle, and allows for studying the mechanism of its pathogenic interaction with plants.

## 5. Conservation of Pseudomonas and Xanthomonas Effector Targets

Both *Pseudomonas* and *Xanthomonas* contain six protein secretion systems of type I, II, III, IV, V, and VI, among which the type III secretion system plays a very important role in their pathogenicity [64]. *Pseudomonas* and *Xanthomonas* can secrete more than 50 effectors with virulence functions into plant cells through the type III secretion system. The study on Pseudomonas effectors found that there was functional redundancy between effectors [68,137], which could be divided into at least two redundant effector groups (REG), different from the classification of *Xanthomonas* effectors described earlier. The first REG consists of AvrPto and AvrPtoB, which act early in *Pseudomonas* infection. AvrPto and AvrPtoB block the function of the PRR complex through inhibition or degradation and then block the expression of downstream components, such as BIK1 to inhibit PAMP-triggered immune (PTI) signaling [138,139]. This is consistent with the role of effectors XopAA, AvrAC, and XopR_Xoo_ in *Xanthomonas* pre-infection pathway. The second REG contains HopM1, AvrE, and HopR1, which interfere with the downstream defense pathway. HopM1 targets vesicle transport by destroying the stability of MIN7, thereby affecting the production of antibacterial compounds [140]. This is similar to the function of the *Xanthomonas* effector XopB_Xe_ which blocks glucose-mediated defense signals by interfering with vesicle trafficking to affect cell-wall-bound convertases [141]. In addition to the same infection pathway, two pathogens also have the same defense signal pathway when they infect plant cells. *Pseudomonas* HopN1 affects ROS production by degrading PsbQ, while *Xanthomonas* XopD_xcc_ interferes with signaling pathways involved in plant defense by preventing ROS production [142,143]. *Pseudomonas* HopAI1 targets MPK4 to interfere with MAPK signal transmission and prevent the activation of effector-triggered immunity (ETI). *Xanthomonas* AvrBsT acts as an ETI inhibitor to prevent the ETI reaction mediated by AvrBs1 and XopQ_Xe_ [144,145]. Thus, in summary, *Pseudomonas* and *Xanthomonas* both need type III effectors to exert virulence and most effectors can play the same or similar role to activate or block plant defense signals. 

## 6. Rhizobial Type III Effector Underlying Symbiosis Establishment

Soil is a complex system containing a large number of pathogenic and symbiotic bacteria that can infect legumes. In particular, rhizobia can form nodules by infecting legumes roots and form a symbiotic organ in which the rhizobia fix nitrogen that can be absorbed by legumes for their growth and development [146]. Due to the high efficiency of the legume plant–rhizobia symbiosis, it is possible to reduce the application of industrial fertilizers, which also makes biological nitrogen fixation important for agriculture and attracts attention from researchers around the world [3]. Nodulation and nitrogen fixation are complex biological processes that require complex signaling between plants and rhizobia. In the presence of flavonoids, rhizobium synthesizes and secretes a lipopylchitosan oligosaccharide, also known as nodulation factor (NF) [147]. To successfully establish rhizobia–legume symbiosis, plant defenses must be suppressed or avoided to allow for rhizobia infection and intracellular accommodation of the symbiont [148]. In addition to NFs, rhizobia produces extracellular polysaccharides, lipopolysaccharides, and in some legume–rhizobia association effectors to facilitate infection in compatible interactions or induce immune responses that block infection in incompatible interactions [146]. 

**Figure 4:**
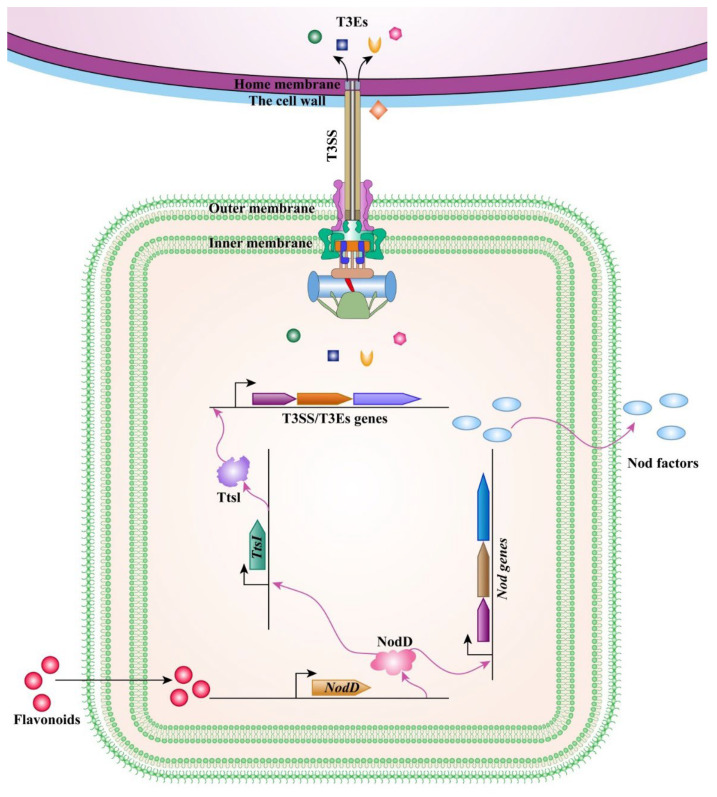
The regulation and expression of type III effectors. Flavonoids released by legume roots trigger the expression of *NodD*. NodD induces the synthesis of Nod factors and the expression of *TtsI*. TtsI then binds to the tts box element of the promoter of T3SS/T3Es genes to regulate the synthesis of T3SS/T3Es.

In soybean, rhizobia also uses T3SS to facilitate root infection [146,149]. The flavonoids secreted by the host roots can be recognized by rhizobia NodD to activate the expression of *Nod* genes but also the *TtsI* expression. TtsI acts as a transcriptional activator, binds to the *tts box* sequence at the promoter of T3SS-related genes, and activates their transcription (Figure 4) [3,150]. These effector proteins can interact with plant immune signaling components and regulate symbiotic interaction [151]. The T3SS secretory machinery is a syringe-like structure composed of approximately 19 different proteins and there is a needle-like structure connecting the rhizobia to the legume cells targeted for multiple type III effectors’ secretion (Table 1). The structural components of rhizobium T3SS have been revealed, by comparing with pathogens, such as *Salmonella* and *Yersinia*. Indeed, proteins that form the basal bodies of rhizobia and pathogen T3SSs share a high degree of conservation [149,150,152]. Unlike pathogens, RhcC1 and RhcC2 components are specific for rhizobia [153]. In addition, the length of the T3SS needle (about 40–80 μm) in rhizobia is significantly longer than the 2 μm of the pathogen ones. This longer needle might be more conducive to secrete effectors across the cell wall of the host cell [149,152,154]. Among *Sinorhizobium fredii* effectors, there is a class of proteins, including NopA, NopB, and NopX, that cannot be secreted. It has been suggested that NopA and NopB pilins are part of the extracellular filament, whereas NopX is also found in extracellular structures but its function seems to be related to the translocation of proteins to the interior of the host cell [155,156,157,158]. Unlike *S. fredii*, some *Bradyrhizobia* with functional T3SSs, instead of containing NopX, contain NopE and NopH, but still have a whole structure of T3SS and can secrete effectors normally [157].

NopJ and NopZ can be considered as putative effectors and NopC, NopD, NopI, NopL, NopM, NopP, NopT, InnB, ErnA, and NopAA are secreted into the host cells. NopC, NopI, NopL, NopP, and ErnA are specific for rhizobia, while NopD, NopJ, NopM, Not, and NopAA are similar to effectors found in different animal and plant pathogens [146,149,151]. These secreted effectors can be recognized by some host cell proteins and are involved in the regulation of symbiosis [149,159]. 

The secreted effector NopC was first identified in *S. fredii* strain HH103 (HH103), is induced by genistein, and is regulated by TtsI [160]. NopC is secreted into soybean cells through the T3SS and the absence of NopC does not affect the synthesis and secretion of other type III effectors, suggesting that NopC is a purely secretory protein [160]. NopC plays an important role in determining host specificity. While HH103 does not nodulate *Lotus japonicus*, the NopC mutant, HH103ΩNopC, can establish a symbiotic relationship with *Lotus japonicus Gifu* and form normal nodules [13]. In soybean, NopC mutation inhibits rhizobia infection by reducing infection thread number. In contrast, overexpression of *NopC* promotes an increased nodule number and weight in soybean roots [161]. During rhizobia infection, NopC activates the expression of *GmCRP* to promote nodulation. NopC-*GmCRP* can interfere with soybean immunity by affecting the expression of *GmPR1* and *PR2* [161]. These findings show that NopC plays an important role in rhizobia infection, possibly by affecting the immunity during nodulation.

NopD was first detected in culture supernatants of *S. fredii* strain HH103. It was induced in the presence of genistein [162] through the action of TtsI [14]. Similar to XopD, one type III effector in *Xanthomonas*, the C-terminal region of NopD contains a domain with homology to the ubiquitin-like protease Ulp1 and can process small ubiquitin-related modifier (SUMO) proteins and cleave SUMO-conjugated proteins [163]. XopD can specifically interact with MYB30, resulting in the inhibition of the transcriptional activation of MYB30 VLCFA-related target genes and in the suppression of immunity in *Arabidopsis* to promote infection of *Xanthomonas* [164]. XopD could also interact with SlERF4, to deSUMOylate SlERF4 to repress ethylene induced-gene expression required for anti-*Xanthomonas* immunity [110]. In rhizobia, NopD positively regulate symbiosis, as NopD mutants produce less nodules. The soybean NopD-interacting proteins have not been identified yet. However, NopD expressed in planta is targeted to nuclei where it accumulates in nuclear bodies [163]. In addition, NopD activity induces ETI-like plant responses, namely cell death, in tobacco [165]. In soybean, *FBD/LRR* and *PP2C* genes were identified by QTL mapping using wild-type and NopD mutants, showing that this effector can suppress their expression during HH103 infection [165]. Further identification and clarification of the host genes involved in interactions with rhizobial effector molecules could enhance the understanding of the soybean–rhizobium symbiosis. Because NopD is targeted to plant nuclei and shows similarity with XopD, we hypothesize that NopD could interact with a transcription factor in soybean and regulate symbiotic nodulation by affecting the immunity pathway in soybean.

NopP is a specific rhizobial effector, was first identified in *Rhizobium sp*. strain NGR234 [166], and is a substrate for host cell kinases. NopP can inhibit strain NGR234 infection and nodule formation in *Flemingia congesta* and *Tephrosia vogelii* [166]. NopP in *S. fredii* HH103 can modify *GmPR1* expression during the HH103 infection of soybean. Earlier studies showed that *GmPR1* is a member of a protein family that includes enzymes (e.g., chitinase and β-1,3-glucanase) that can directly attack pathogen structures, thereby acting as an antimicrobial determinant [167,168]. During HH103 infection, NopP could induce the *GmMAPK3* and *TLP* gene expression, to promote nodulation in soybean Charleston [168]. Further, in soybean, *Rj2* can restrict the nodulation involving specific rhizobial strains, such as *Bradyrhizobium diazoefficiens* USDA122 [169], suggesting that NopP could interact with Rj2 to mediate the incompatibility between rhizobia and soybean [12]. Through genetic analysis of soybean natural varieties, the soybean gene *GmNNL1* was shown to be involved in symbiosis. By interacting with NopP from USDA110, *GmNNL1* could trigger immunity and suppress rhizobia infection [170].

The type III effector NopL was first identified in *Rhizobium sp*. strain NGR234. It is a rhizobia-specific effector protein with multiple phosphorylation sites. In addition to the N-terminal secretory signal sequence, NopL consists of two large repeats and a C-terminal domain [171]. Its mutation can significantly reduce nodule formation in *Phaseolus japonicum* and accelerate the senescence of *Phaseolus japonicum* nodules [172]. In addition, the expression of NopL in tobacco and rhizome can inhibit the expression of disease-progression-related proteins, such as class I chitinase and class I glucanase, indicating that NopL is interfering with the immune response of plants. NopL localizes to the plant nucleus of onion cells and is phosphorylated at multiple sites by SIPK (SA-activated MAP kinase), a tobacco MAP-kinase. Although its phosphorylation has not been verified in legumes, it can be predicted that NopL will be hyperphosphorylated by legume MAP kinases [173]. In soybean, the secretion of NopL into the host promotes rhizobia infection and nodule formation. In the presence of NopL, the *PP2C* (protein phosphatase 2C family)*-related* gene and *RPK* are induced in soybean, but their functions have not been further studied [174].

Similar to NopD, NopM was first detected in culture supernatants of *S. fredii* strain HH103 [162]. It is a member of the IpaH (invasion-plasmid antigen H) effector family that is present in various bacteria, such as *Shigella flexneri*, *Salmonella enterica, and Ralstonia solanacearum* [175,176]. RipAW and RipAR, two type III effectors of this family in the plant pathogen *R. solanacearum*, significantly suppress pattern-triggered immunity (PTI) responses, such as the production of reactive oxygen species and the expression of defense-related genes, when expressed in leaves of *Nicotiana benthamiana* [177]. RipAW and RipAR probably suppress host PTI responses by its E3 ubiquitin ligase activity [177]. NopM, such as RipAW, RipAR, and other IpaH family effectors, possess a variable N-terminal domain containing leucine-rich repeats (LRR domain) and a conserved C-terminal E3 ubiquitin ligase (NEL) domain. When expressed in *N. benthamiana*, NopM of strain NGR234 represses the production of reactive oxygen species induced by flagellin [175]. Studies using NopM showed that it functions as an E3 ubiquitin ligase, which formed polyubiquitin chains in vitro [175,176]. Analysis of a mutated NopM protein indicated that the cysteine at position 338 is required for E3 ubiquitin ligase activity. Furthermore, NopM could be localized to the plant nucleus of onion cells, suggesting that NopM might ubiquitinate nuclear proteins [175,176]. Expression of NopM in yeast indicated that NopM expression also impaired MAP kinase signaling of mating pheromone response pathway [176]. Further experiments indicated that serine residue 26 of NopM is phosphorylated in plants and that NopM can be phosphorylated by the salicylic-acid-induced protein kinase (NtSIPK), a mitogen-activated protein kinase (MAPK) of tobacco. Hence, NopM is a phosphorylated T3SS effector that can interact with itself, with ubiquitin, and with MAPKs [175,176]. Mutant analysis showed that the absence of NopM in NGR234 suppresses symbiosis with the host plant *Lablab purpureus* [175]. Nodulation tests with the C338A point mutation showed that the E3 ubiquitin ligase activity of NopM is required for optimal nodulation of *Lablab purpureus* and has a phenotype similar to the NGR234 mutant [175]. Although the studies in other organisms not interacting with rhizobia can provide a certain basis for analyzing the role of NopM, the MAPK protein interacting with NopM in legumes has not been verified yet and further studies are needed to identify its function in legumes.

NopT is an effector protease belonging to the AvrPphB effector family. The protease activity of this family is dependent on a catalytic triad of amino acids that is present in NopT [178]. In *P. syringae*, AvrPphB mutation promotes the growth of *P. syringae* in host leaves [179,180]. AvrPphB cleaves AtPBS1 to cause a conformational change that activates AtRPS5 and triggers ETI-related downstream responses to inhibit *P. syringae* infecting *A. thaliana* [82,179,180]. NopT from rhizobia can translocate into plant cells and localize to the plant plasma membrane. Expression of NopT in tobacco induces death of the leaves [178]. Mutation of NopT in *Mesorhizobium amphore* restores nodulation of the legume tree black locust. Compared with the wild strain, this NopT mutant induces black locust roots to generate more jasmonic acid, salicylic acid, and hydrogen peroxide [181]. Similarly, in NGR234, the protease activity of NopT negatively affects the nodulation of smooth crotalaria. When the USDA257 strain that contains a mutated NopT gene expresses the NopT gene of NGR234, it induces considerably fewer nodules in soybean [178]. Similar to AvrPphB, NopT could cleave GmPBS1 proteins to an active form that suppress nodulation in soybean, indicating that activation of a GmPBS1-1-mediated resistance pathway impairs nodulation in soybean [178]. The comparison of the effects of AvrPphB and NopT on *P. syringae* infection and symbiosis, respectively, suggests that legumes have evolved immune responses that are somewhat similar with pathogens and rhizobia. It also shows an evolutionary contradiction or balance between the need to resist pathogen infections, on the one hand, and the need to promote the establishment of symbiosis on the other.

NopAA is an effector belonging to the Glycoside hydrolase 12 (GH12) family. It can be secreted into the extracellular environment and its expression is promoted by genistein [182]. The enzyme activity of NopAA was investigated in vitro, using xyloglucan and β-glucan as substrates. NopAA hydrolyzes both xyloglucan and β-glucan directly to sugars. Both xyloglucan and β-glucan are important components in cellulose and hemicellulose in plant cell walls and their hydrolysis could promote the entry of rhizobia into host cells [183]. In *Phytophthora sojae*, one glycoside hydrolase, PsXEG1, was identified as a glycoside hydrolase 12 family member and can hydrolase the same substrates as NopAA [184]. Because PsXEG1 (pathogen-secreted apoplastic xyloglucan-specific endoglucanase) is an effector of *P. sojae*, the growth of *P. sojae* could be promoted using a PsXLP1 decoy that was similar to PsXEG1 but without its enzymatic activity. Additionally, PsXLP1 (PsXEG1-like protein) can protect PsXEG1 from GmGIP1 (PsXEG1 interacting protein) binding in vitro and in plants [184,185]. Mutation of NopAA suppresses nodulation by inducing fewer infection threads and reducing the nodule number [183]. Using genetic approaches, *GmARP* (NopAA-related protein) was shown to positively regulate the establishment of symbiosis after interaction with NopAA. Similarly, *GmARP* overexpression promotes nodule formation after inoculation with either the wild strain or the NopAA mutant. In contrast, in *GmARP*-silenced plants, no differences in nodule number or dry weight were observed between plants inoculated with HH103 or the corresponding NopAA mutant [183]. These results suggest that *GmARP* is a positive regulator of nodule formation and that it mediates NopAA signaling in plants. Thus, in addition to the hydrolase activity of NopAA, NopAA may act as a signaling molecule with *GmARP* to regulate symbiotic nodulation. 

In future studies, more and more type III effectors will be identified and studied in the frame of symbiosis. Additional type III effectors might be identified and might participate in nodulation using different mechanisms and might unravel other connections with the immune signaling pathway of host cells. Recent studies have shown that the type III secretion system and the type III effectors of rhizobia and pathogens are conserved, indicating a probable common origin. This conservation is also an important tool to decipher their action during symbiosis. The identification and functional analysis of interacting proteins of these type III effectors in the legume host are very important for understanding their mode of action in relation to immunity. 

## 7. Conclusions and Perspectives

The findings summarized in this review point to an increasing interest in the identification and determination of the functions of bacterial and nematode secreted effectors. Dozens of different soybean germplasms, including cultivars, landrace, and wild soybean, have been tested to determine the symbiotic and pathogenic responses and identify these secreted effectors. More and more scientists are now paying attention on the functional analysis of numerous secreted effectors and the signaling they trigger. Once the detailed function of different secreted effectors is clear, breeders could utilize these results to assist cultivar breeding. Fine regulation of the nodule number and nitrogen fixation efficiency are desired targets for symbiosis application in the field. In addition, pathogen and symbiotic effector targets participate in common signaling pathways. Thus, one may use gene-modification strategies to regulate the soybean responses to both rhizobium and pathogens, including leaf pathogens, if signaling includes the shoot and the root. Therefore, more efforts should be made to increase our knowledge about soybean responses to beneficial and pathogenic microbes and cluster these genes on new cultivar breeding. Doing this, we could reduce the use of nitrogen fertilizers and chemical pesticides. 

## Figures and Tables

**Table 1:** Core structural components and functions of T3SSs and T3Es in rhizobia.

No.	Composition	Function	Target in Host Cells	Effect of Mutation on Nodule
	Structural Components of T3SS			
1	RhcN	ATPase	-	Nod^−^
2	NolV	Stator	-	-
3	RhcQ	Cytoplasmic ring	-	-
4	RhcO	Stalk	-	-
5	RhcV	Export gate	-	-
6	RhcU	Autoprotease	-	-
7	RhcR	Inner membrane component	-	-
8	RhcS	Inner membrane component	-	-
9	RhcT	Inner membrane component	-	-
10	RhcJ	Inner membrane ring	-	-
11	RhcD	Inner membrane ring	-	-
12	RhcC1	Secretin, outer membrane ring	-	-
13	NolU	Inner rod	-	Nod^−^
14	RhcC1	Secretin, outer membrane ring	-	Nod^−^
15	NopA	Needle	-	Nod^−^
16	NopB	Needle	-	Nod^−^
17	NopX	Translocation pore	-	Nod^−^ and Delayed nodulation
18	NopE	Translocation pore	-	-
19	NopH	Translocation pore	-	-
	Secretable effector of T3Es			
20	NopC	Suppress immunity	Unknown	Nod^−^
21	NopD	DeSUMOylation	Rj4	Nod^−^
22	NopL	Substrate for plant kinase	SIPK	Nod^−^
23	NopJ	Putative acetyltransferase	Unknown	Nod^−^ or Nod^+^
24	NopM	E3-ubiquitin ligase	Unknown	Nod^−^
25	NopP	Substrate for plant kinase	NNL1	Nod^−^
26	NopT	Cysteine proteases	PBS1	Nod^−^ or Nod^+^
27	NopZ	Putative effector	Unknown	-
28	InnB	Unknown	Unknown	Nod^−^
29	NopAA	Cellulase	Unknown	Nod^−^
